# Impact of Bariatric Surgery on the Stability of the Genetic Material, Oxidation, and Repair of DNA and Telomere Lengths

**DOI:** 10.3390/antiox12030760

**Published:** 2023-03-21

**Authors:** Franziska Ferk, Miroslav Mišík, Benjamin Ernst, Gerhard Prager, Christoph Bichler, Doris Mejri, Christopher Gerner, Andrea Bileck, Michael Kundi, Sabine Langie, Klaus Holzmann, Siegfried Knasmueller

**Affiliations:** 1 Center of Cancer Research, Medical University of Vienna, Borschkegasse 8a, 1090 Vienna, Austria; 2 Department of Surgery, Medical University Vienna, 1090 Vienna, Austria; 3 Department of Analytical Chemistry, Faculty of Chemistry, University of Vienna, 1090 Vienna, Austria; 4 Joint Metabolome Facility, University and Medical University Vienna, 1090 Vienna, Austria; 5 Department for Environmental Health, Center of Public Health, Medical University of Vienna, 1090 Vienna, Austria; 6 Department of Pharmacology & Toxicology, School for Nutrition and Translational Research in Metabolism (NUTRIM), Maastricht University, 6229 ER Maastricht, The Netherlands

**Keywords:** bariatric surgery, DNA stability, DNA repair, redox status, proteome profiling

## Abstract

Obesity causes genetic instability, which plays a key-role in the etiology of cancer and aging. We investigated the impact of bariatric surgery (BS) on DNA repair, oxidative DNA damage, telomere lengths, alterations of antioxidant enzymes and, selected proteins which reflect inflammation. The study was realized with BS patients (*n* = 35). DNA damage, base oxidation, BER, and NER were measured before and 1 month and 6 months after surgery with the single-cell gel electrophoresis technique. SOD and GPx were quantified spectrophotometrically, malondealdehyde (MDA) was quantified by HPLC. Telomere lengths were determined with qPCR, and plasma proteome profiling was performed with high-resolution mass spectrophotometry. Six months after the operations, reduction of body weight by 27.5% was observed. DNA damage decreased after this period, this effect was paralleled by reduced formation of oxidized DNA bases, a decline in the MDA levels and of BER and NER, and an increase in the telomere lengths. The activities of antioxidant enzymes were not altered. Clear downregulation of certain proteins (CRP, SAA1) which reflect inflammation and cancer risks was observed. Our findings show that BS causes reduced oxidative damage of DNA bases, possibly as a consequence of reduction of inflammation and lipid peroxidation, and indicate that the surgery has beneficial long-term health effects.

## 1. Introduction 

According to the WHO, 1.9 billion adults are overweight and 650 million are obese. Furthermore, the organization stated that excess body weight (BW) causes around 2.8 million deaths annually [1].

A most promising strategy to reduce adverse health effects in individuals with severe obesity is bariatric surgery (BS), which leads to weight loss and reduction of the incidence of weight-related disorders, including diabetes type II, cardiovascular diseases, and cancer [2,3,4]. The latest report of the International Federation for Surgery of Obesity and Metabolic Disorders contains data from 50 countries and states that 507,298 operations were performed in 2021; according to the American Society for Metabolic and Bariatric Surgery, the number of BS increased substantially in the last years (ASMBS 2021, accessed on 10 December 2021, www.asmbs.org). 

Different BS techniques have been developed, and the most frequently used procedures are gastric sleeve (GS) and Roux-en-Y gastric bypass (RYGB), one-anastomosis gastric bypass (OAGB), and gastric band. It was postulated that OAGB reduces the operation time and early and late complications [5]. It is well documented in systematic reviews that BS improves the health status of overweight individuals, i.e., it normalizes glucose metabolism, reduces the risk for CVD and diabetes, and increases the lifespan [6,7,8].

Only a few studies have been published which indicate that overweight and obesity lead to DNA damage, which plays a key role in the etiology of several diseases, including cancer as a consequence of inflammation and release of radical oxygen species [9]. The aim of the present study was a comprehensive investigation of the consequences of weight loss of BS patients (*n* = 35) who underwent different types of surgery on genomic and telomeric stability, oxidative damage of DNA bases, DNA repair, and parameters which have an impact on the integrity of the genetic material (redox status, proteins, which reflect inflammation). The design which we used was identical to that of earlier dietary intervention trials, i.e., the extent of DNA damage and other parameters are monitored before the surgery and at two time points (1 and 6 months) after the operations. 

DNA damage, oxidation of purines, and DNA repair (nucleotide excision repair, NER and base excision repair, BER) were measured with different protocols of the single-cell gel electrophoresis (SCGE) assay. This method is based on the quantification of DNA migration in an electric field [10] and is increasingly used in human biomonitoring [11]. The endpoints, which are measured in SCGE experiments are related to human health. It was found that the extent of comet formation predicts the risk of mortality [12]. Additionally, it is well documented that DNA migration is increased in patients with high-prevalence diseases, including specific forms of cancer [13]. Oxidation of DNA bases is a consequence of inflammation and redox stress, and it was stated by the European Food Safety Authority (EFSA) that prevention of oxidative damage has a positive impact on human health [14]. DNA repair systems (BER and NER) play a causal role in the etiology of cancer and other diseases [15,16]. Telomere shortening causes cellular senescence [17], and evidence is accumulating that it may accelerate aging processes in humans [18]. Superoxide dismutase (SOD) and glutathione peroxidase (GPx), which were monitored in the present study are antioxidant enzymes which reflect the redox status [19]; low activities are associated with different human pathologies [20,21]. Malondialdehyde (MDA) is a lipid peroxidation (LP) product which reflects the oxidation of fatty acids and causes damage to the genetic material [22,23].

Only a few studies have been realized in which the consequences of BS on DNA stability were investigated. All earlier trials were performed with patients who underwent GS and RYGB operations, while no data are currently available concerning OAGB surgery. Bankoglu and co-workers investigated the consequences of BS on DNA damage in SCGE experiments [24,25]. Furthermore, some studies have been published concerning the formation of oxidized guanine (8-oxoGuo and 8-OHdG) [26,27,28]. The impact of BS on BER and NER has not been studied according to our knowledge, but Habermann and co-workers investigated DNA repair in obese postmenopausal women after weight loss [29]. Results of studies concerning the reduction of telomere lengths after BS are controversial; some of them point in the direction of beneficial long-term effects [30], also the findings concerning alterations of the activities of antioxidant enzymes after weight loss are inconsistent [28,31,32].

## 2. Methods

### 2.1. Recruitment of the Participants 

The study was approved (18.10.2016) by the Ethical Committee of Medical University of Vienna (1479/2016). All patients provided written consent, and 40 patients were recruited from the Department of Surgery, MUW; 35 individuals finished the study. Inclusion criteria were BMI values > 35 kg/m^2^ and an age range between 18 and 60 years. Exclusion criteria were chronic diseases (diabetes mellitus type II, cystic fibrosis, arthritis, asthma), intake of food supplements before surgery, and intake of anti-inflammatory drugs and pharmaceuticals with antioxidant properties. 

The patients underwent different types of BS, namely RYGB (*n* = 11), OAGB (*n* = 19), GS (*n* = 2) and SADI-S (*n* = 3). The study had an intervention design, i.e., values that were obtained before the surgery were compared with the values that were obtained after the operation. 

**Figure 1:**
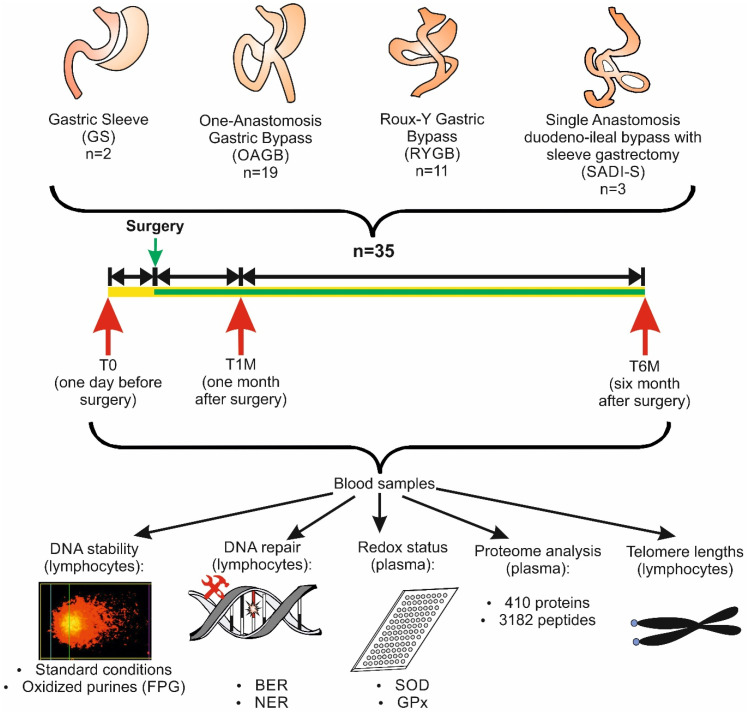
Schematic presentation of the study design. Abbreviations: BER, base excision repair; FPG, formamidopyrimidine glycosylase; GPx, glutathione peroxidase; NER, nucleotide excision repair; SOD, superoxide dismutase.

The same design was used in many other dietary studies [33,34] and in weight loss trials [9,25]. Blood samples (40 mL/patient) were collected at three time points, namely one day before the operation (T0) and 1 month (T1) and 6 months (T2) after the surgery (Figure 1).

BS leads to nutritional deficiencies [35]; therefore, all participants consumed a supplement containing vitamins and trace elements after the surgery (WLS Forte, Berlin, Germany). The composition is specified in Table S1.

### 2.2. Isolation of Plasma and Lymphocytes

Plasma was separated from blood by centrifugation (650 g, 20 min). Subsequently, aliquots were stored at −80 °C. Peripheral lymphocytes were isolated by gradient centrifugation (800 g, 15 min, 16 °C) with Histopaque (Sigma–Aldrich, Steinheim, Germany). The pellets were suspended in 100 μL RPMI and aliquoted in Biofreeze Medium (Biochrom AG, Berlin, Germany, frozen overnight at −80 °C and stored in liquid nitrogen.

### 2.3. SCGE Experiments with Lymphocytes 

The experiments were carried out according to international guidelines for SCGE experiments [10,36]. The viability of the cells was determined by use of a CASY cell counter (Schärfe-System GmbH, Reutlingen, Germany); DNA damage was only analyzed in samples with a viability ≥ 70% since reduced viability may cause misleading results [37].

For standard comet assays the cells were mixed with 0.5% LMPA and transferred to agarose coated slides (1.0% NMPA). After lysis (pH 10.0), electrophoresis was carried out under alkaline conditions (30 min, 300 mA, 1.0 V/cm, at 4 °C, pH > 13). Subsequently, the slides were washed two times (8 min), air-dried, and stained with propidium iodide (10.0 μg/mL, Sigma-Aldrich, Steinheim, Germany). 

Per experimental point, three slides were made and 50 cells were evaluated randomly. Cells were examined under a fluorescence microscope (Nikon EFD-3, Tokyo, Japan) using a 20-fold magnification. DNA migration was determined with a computer aided image analysis system (Comet Assay IV, Perceptive Instruments, Bury Saint Edmunds, UK). The percentage of DNA in tail (% DNA) was monitored as an endpoint, as suggested in international guidelines [37].

To determine the formation of oxidized purines, nuclei were exposed after lysis (1 h) to formamidopyrimidine glycosylase (FPG, Sigma-Aldrich, Steinheim, Germany). To establish the optimal enzyme concentration, a calibration experiment was carried out [38]. After lysis, the slides were washed twice with enzyme reaction buffer (pH 8.0, 8 min.). Subsequently, the nuclei were treated (30 min., 37 °C) with 50 μL of FPG solution (1:3000 dilutions) or with the enzyme reaction buffer. After the treatment, electrophoresis was carried out and the slides were evaluated as described above. To calculate the extent of DNA damage attributable to formation of oxidized purines the values, which were obtained with the enzyme buffer were subtracted from the values, which were obtained with the lesion specific enzyme [38]. Technical controls (from two individuals, who were not involved at the study) were included in all experiments [13].

### 2.4. Measurement of BER and NER

A modification of the SCGE assay was used to measure BER and NER [10]. This approach is based on the ability of repair proteins in cell extracts to recognize and to cut substrate DNA, which contains specific lesions [36]. 

Protein extracts were prepared from lymphocytes (1.5 × 10^6^) by centrifugation (700 g, 10 min, 4 °C) after addition of 65 μL of extraction buffer (45 mM HEPES, 0.4 M KCl, 1 mM EDTA, 0.1 mM dithiothreitol, 10% glycerol, pH 7.8) with 1% of Triton X-100 (Buffer A). Samples were vortexed at top speed and snap-frozen in liquid nitrogen. Lysates were thawed and centrifuged at 15,000× *g* (5 min at 4 °C). Supernatants (55 μL) were collected and mixed with 220 μL cold buffer B (40 mM HEPES, 0.5 mM EDTA, 0.2 mg/mL BSA, 0.1 M KCl, pH 8.0). Protein concentrations of extracts were quantified with a DC Protein Assay Kit (BIO-RAD, Veenendaal, The Netherlands).

A549 cells (a human lung fibroblast carcinoma line, provided from the ATCC, Manassas, VA, USA) were used as substrate cells. They were cultivated in RPMI 1640 medium (low glucose, with L-glutamine), supplemented with 10% FCS and U/mL penicillin/streptomycin (Invitrogen, Darmstadt, Germany) under humidified conditions (5% CO_2_, 37 °C). At 85–90% confluence, the cells were washed with Dulbecco’s PBS and harvested with 0.25% trypsin-EDTA.

For the BER measurements, a photosensitizer, Ro 19-8023 (Chiron AS, Trondheim, Norway) at 1.0 μM was used, which causes oxidation of DNA bases. Substrate cells were treated in presence and absence of visible light (400 W, 60 cm distance, 4 min). Subsequently, they were centrifuged (700× *g* for 10 min). Subsequently, the pellets were re-suspended in freezing medium (Biofreeze Medium, Biochrom AG, Berlin, Germany) and cryopreserved at −80 °C. For the NER assay, UVC (2.0 Jm^−2^, 22 s. on ice) was used to produce cyclobutane pyrimidine dimers.

After the chemical treatments, the cells (2.5 × 10^4^ per gel) were embedded in agarose and lysed. For NER measurements. The slides were washed twice for 10 min buffer N (45 mM HEPES, 0.25 mM EDTA, 0.3 mg/mL BSA, 2% glycerol, pH 7.8) and for BER in buffer B (40 mM HEPES, 0.5 mM EDTA, 0.2 mg/mL BSA, 0.1 M KCl, pH 8.0). Subsequently, the nuclei were incubated either with 40 μL “extract mix” (lymphocyte extract, extract buffer with Triton X-100 and reaction buffers) or with control buffer. Alkaline unwinding (40 min) and electrophoresis (30 min) were performed as in standard comet experiments.

### 2.5. Measurement of GPx and SOD 

The activities of GPx and SOD were measured spectrophotometrically (Tecan Infinite M200 Plate Reader, Switzerland) with commercially available kits (GPx, ab102530; SOD, ab65354, Abcam, Cambridge, UK) according to the instructions of the manufacturers at 350 nm for GPx and at 450 nm for SOD. All samples were measured in duplicates. SOD activity was measured as % inhibition of formation of a water-soluble tetrazolium salt.

### 2.6. Measurement of Malondiadehlyde in Plasma 

MDA concentrations were determined in plasma according to the method of Ramel et al. [39], which we used in earlier studies [40,41,42]. The samples were neutralized after heating (60 min, 100 °C) with methanol/NaOH and centrifuged at 3000 rpm (for 3 min). Subsequently, MDA was measured with HPLC with excitation at 532 nm and emission at 563 nm (LaChrom Merck Hitachi Chromatography system, Tokyo, Japan). Each sample was analyzed in duplicate.

### 2.7. Measurement of the Telomere Lengths 

Genomic DNA was isolated from pellets using Gentra PureGene Cell Kit (Qiagen, Venlo, Netherlands). Quantification of DNA was conducted with iQuant Broad Range dsDNA Quantification Kit (Genecopoeia, Rockville, MD, USA), according to the protocol of the manufacturer with a Qubit Fluorometer (Thermo Fischer Scientific Inc., Waltham, MA, USA) and stored at −80 °C for further measurements.

Telomere lengths were determined by monochrome multiplex qPCR as described by Cawthon [43]. Telomeric contents were measured in reference to one selected experimental DNA sample and to one of the single-copy genes 36B4 and ALB; for more details see [44]. The relative telomere-to-single-copy-gene (T/S) ratio was determined in each sample in triplicate.

### 2.8. Proteome Analyses of the Plasma Samples

The protein concentrations of the plasma samples were determined with a bicinchoninic acid (BCA) assay. Enzymatic digestion of samples was achieved applying a protocol using the S-trap technology [45]. Finally, peptides were eluted, dried and stored at −20 °C until LC-MS/MS analyses.

For LC-MS/MS analyses, dried peptide samples were reconstituted in 10 μL of 30% formic acid (FA) containing 4 synthetic standard peptides (10 fmol/μL) and further diluted with 80 μL mobile phase A (97.9% H_2_O, 2% ACN, 0.1% FA). LC-MS/MS analyses were performed on a Dionex Ultimate 3000 nano LC-system coupled to a timsTOF Pro mass spectrometer (Bruker) equipped with a captive spray ion source. For mass spectrometric analyses, the timsTOF Pro Mass Spectrometer (Bruker Daltonics USA, Billerica, MA, USA) was operated in the parallel accumulation-serial fragmentation (PASEF) mode. Trapped ion mobility separation was achieved by applying a 1/k0 scan (0.60–1.60 V. s/cm^2^) resulting in a ramp time (166 ms). All experiments were performed with 10 PASEF MS/MS scans per cycle leading to a total cycle time (1.88 s). MS and MS/MS spectra were recorded using a scan range (*m*/*z*) from 100 to 1700.

Protein identification and label-free quantification (LFQ) were carried out using MaxQuant (version 1.6.17.0, Max-Planck-Institute of Biochemistry, Martinsried, Germany) running Andromeda as search engine and searching against the SwissProt Database (version 14122019 with 20,380 entries, SIB Swiss Institute of Bioinformatics, Lausanne, Switzerland); for details, see Cox und Mann [46]. Search criteria included an allowed peptide tolerance for the first and main search of 20 and 10 ppm and a maximum of 2 missed cleavage sites. All peptide and protein identifications met a false discovery rate (FDR) ≤0.01. After protein identification, proteins were filtered for common contaminants as well as reversed sequences, and data evaluation was performed using Perseus (version 1.6.1.3, Max-Planck-Institute of Biochemistry, Martinsried, Germany).

### 2.9. Statistical Analyses 

SCGE data were arcsine transformed, and telomere lengths were log transformed to obtain homogenous variances and normality of residuals. The Box M test was carried out to assess symmetry of the variance-covariance matrices. Kolmogorov–Smirnov tests with Lilliefors’ corrected *p*-values were performed to assess normality of residuals. For the analysis of the different endpoints of DNA stability and repair, telomere lengths, and enzyme activities, a general linear model was applied with age, sex, smoking status, and BMI at baseline as covariates. Testing against baseline values was performed by linear contrasts with Bonferroni correction. In addition, trend tests with respect to time since surgery were performed. All analyses were performed by Stata 13.1 (StataCorp, College Station, TX, USA). Graphs were prepared by GraphPad Prism 5.0 (Graphpad Software, San Diego, CA, USA). 

Statistical analysis of plasma proteomics data was performed using a software package (version 1.6.1.3, Max-Planck-Institute of Biochemistry, Martinsried, Germany). Prior to the analysis, LFQ intensity values were transformed (log2x). T-tests were performed between the study groups applying an FDR of 0.05 and a S0 of 0.1, whereby S0 controls the relative importance of *t*-test *p*-value and difference between the means. Results are shown as volcano plots.

## 3. Results 

### 3.1. Description of the Study Group

The demographic characteristics of the study group are summarized in Table 1. The average BMI values were in all group similar and the body weights were also in a narrow range. About one-third of the participants were females.

### 3.2. Impact of BS on Weights and BMIs

**Figure 2:**
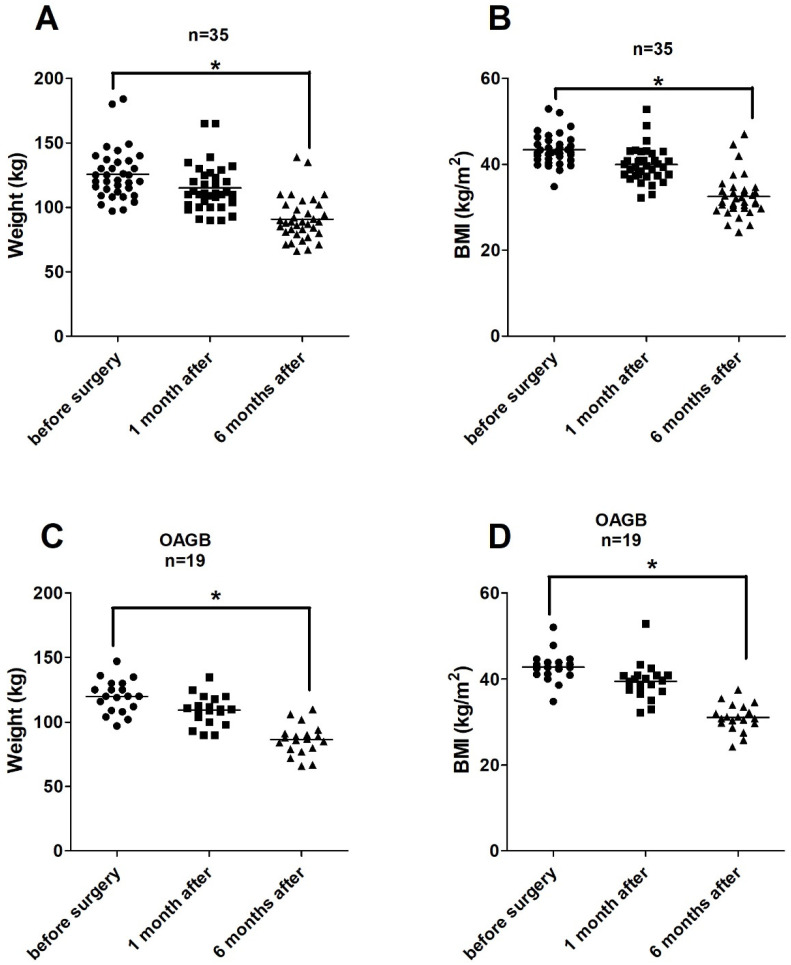
(**A**–**D**). Alterations of the body weights and of the BMI values after BS in all patients (2**A**,**B**, *n* = 35) and in the OAGB group (2**C**,**D**, *n* = 19). Points indicate individual data. Asterisks indicate statistical significance (* *p* ≤ 0.05).

Figure 2A–D show the reduction of the body weights and BMIs in the overall BS group. The values decreased substantially (*p* < 0.001) 6 months after the surgery (BWs by 27.5% and BMIs by 28%). The decline in both parameters was similar in the OAGB group (Figure 2C,D). 

### 3.3. Impact of BS on DNA Stability and Oxidative DNA Damage and Repair

**Figure 3:**
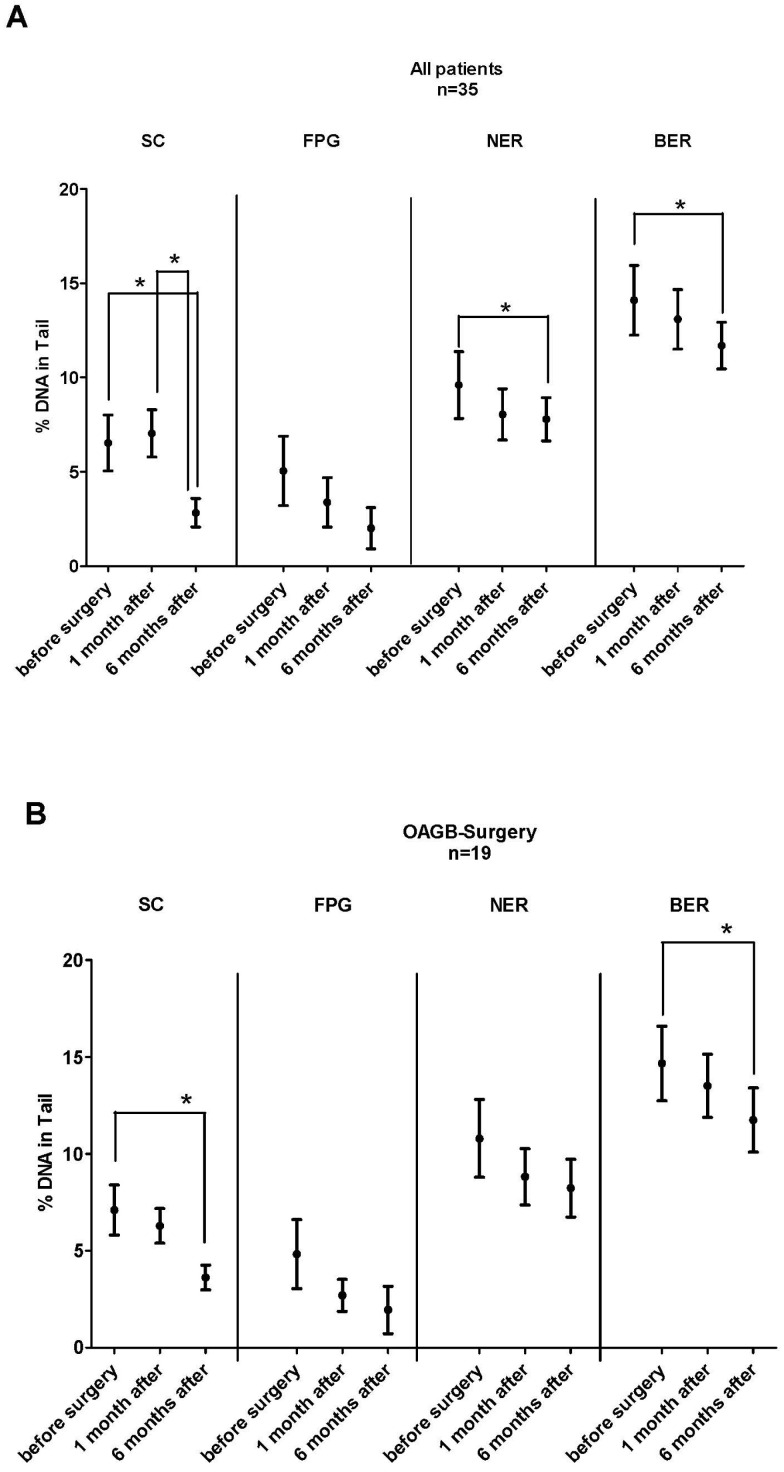
(**A**,**B**). Impact of BS on DNA stability, oxidative DNA damage, and DNA repair capacity in all BS patients (3A, *n* = 35) and in patients who underwent OAGB surgery (3B, *n* = 19). The experiments were conducted under standard conditions (SC) and after treatment of the nuclei with FPG. BER and NER activities were measured after pre-damage of the nuclei with cytosolic extracts of lymphocytes from patients that were collected at different time points before and after surgery (for details, see Materials and Methods). From each patient, three slides were made in parallel, and 50 cells were analyzed from each slide. Bars represent means ± SEM. Asterisks indicate statistical significance (* *p* ≤ 0.05).

Figure 3A summarizes the results of the SCGE experiments. Significant reduction (*p* = 0.009, 54%) in DNA damage was observed under standard conditions after 6 months. No significant effects were found 1 month after the surgery (scatter plots showing the individual values are shown in Supplementary Figure S1A–D).

The middle section of the graphs shows alterations of the FPG-sensitive sites. The extent of comet formation attributable to formation of oxidized purines declined after the surgery. This effect did not reach significance, but a clear trend was observed (*p* < 0.001).

The activities of BER and NER decreased after 6 months (NER, *p* = 0.049 and BER, *p* = 0.001). A decline in both repair systems was observed already 1 month after the surgery (NER, 16.5% and BER, 7%) but this effect did not reach significance.

We analyzed also the effects in the RYGB subgroup. The findings, which were obtained under standard conditions, were identical to those obtained in the overall group (13% decrease after 1 months and 47% after 6 months). FPG sensitive sites declined by 12.5% after 6 months. We observed an unexpected increase (by 29%) of oxidative purines after 1 month. The activity of BER was reduced by 8% after 1 month and 14% after 6 months. The NER activity declined by 18% after 1 month and by 26% after 6 months. 

Figure 3B shows the results which were obtained with the OAGB patients. The effects were similar to those found in the overall group. Significant changes were detected under standard conditions, which reflect single- and double-strand breaks (*p* = 0.0002, 50%) after 6 months. The activity of BER was clearly reduced after this period (*p* = 0.008), while the decline in oxidized bases showed only a trend (*p* < 0.001). 

### 3.4. Alterations of the Activities of Antioxidant Enzymes

Table 2 and Figure S2A,B show the results of GPx and SOD measurements before and after BS. The activities of both enzymes declined slightly after one (SOD: 6.4% GPx: 7.3%) and 6 months (SOD: 1.9% and GPx: 2.5%) but these effects did not reach significance in the overall group and in the subgroups.

### 3.5. Results of Malondialdehyde Measurements

We found in the overall group evidence of a significant decrease in this LP product in plasma of the patients half a year after the surgery (baseline before surgery 4.42 ± 0.72 μM/L, after 1 month 4.21 ± 1.1 μM/L, and after 6 months 2.60 ± 0.89 μM/L); also, in the subgroups RYGB and OAGB, a clear decline was detected (OAGB: before surgery: 4.49 ± 0.86 μML, after 1 month 4.32 ± 0.90 μM/L, after 6 months 2.67 ± 0.80 μM/L; RYGB: baseline before surgery 4.50 ± 0.57 μM/L, after 1 month 4.24 ± 0.61 μM/L an after 6 months 2.60 ± 0.48 μM/L). 

### 3.6. Alterations of the Telomere Lengths

Table 3 summarizes the results of measurements of the telomere lengths. The T/S values varied over a broad range and increased in the period between one and 6 months in the ALB assay in the overall group and also in OAGB subgroup (*p* = 0.022 and *p* = 0.046). The 36B4 assay showed a similar trend without reaching significance. 

### 3.7. Alterations of the Proteome Profile 

**Figure 4:**
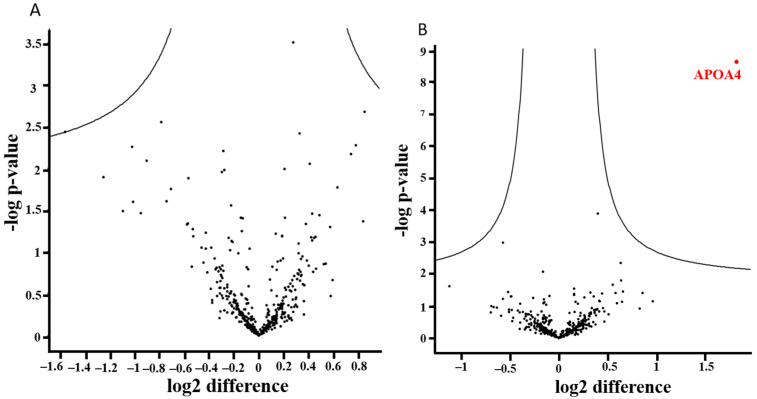

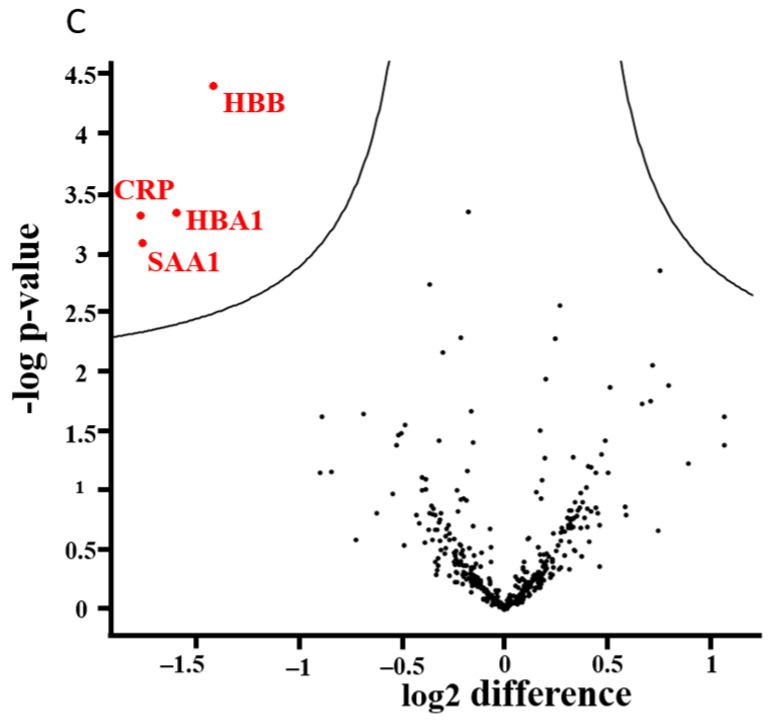
(**A**–**C**). Results of proteomic analyses with plasma samples of the BS patients (*n* = 35): 410 proteins and 3182 peptides were analyzed. The distribution of up- and downregulated proteins is shown in volcano plots. For each identified protein, the fold-changes were plotted on a logarithmic scale to the basis of 2 (ln2Δt-test), and the corresponding *p*-values (−log *p*-value) for individual proteins are indicated.

We analyzed 410 proteins compiled from 3182 peptides. The individual proteins are listed in Supplementary Table S2. Figure 4A–C show the results of analyses with plasma samples. No alterations were observed after 1 month (Figure 4A), but significant effects were found 6 months after the surgery (Figure 4C). Four proteins were downregulated, namely serum amyloid A1 (SAA1), C-reactive protein (CRP), and two hemoglobin subunits (HBB and HBA1). The level of apolipoprotein A-IV (APOA4) was significantly higher 1 month after surgery compared to the level detected 6 months after BS (Figure 4B).

## 4. Discussion

As mentioned in the introduction, several earlier investigations indicated that BS has beneficial health effects. For example, it was found that it affects the artherogenic properties of plasma lipoprotein [47] and reduces cardiovascular risks [48]. Furthermore, several studies showed that it normalizes the metabolism of amino acids and proteins [49] as well as the levels of systemic hormones and signaling peptides [50,51,52,53,54] and improves glycemic control [51]. 

The present study focused on alterations of the stability of the genetic material and related parameters. These observations enable to draw conclusions concerning beneficial long term health effects of the operations. An earlier study focused on the consequences of RYGB and GS, which are the most frequently used techniques. The present study provides additional information about OAGB, which is the most widely used technique after GS and RYGB [55].

We observed a time-dependent weight loss in all participants. Furthermore, we studied for the first time the impact of BS techniques on DNA repair functions. The effects in the different subgroups were more or less identical and similar to findings of earlier studies [56,57,58].

Six months after the surgery, the extent of DNA damage decreased substantially (54%) in the overall group and a similar reduction was found in the OAGB group (50%) and in patients after RYGB (47%). Only one study with BS patients who underwent RYGB and GS [25] has been published in which DNA damage was analyzed in SCGE experiments with lymphocytes and whole blood from the same patients [24,25]. The authors did not detect reduced comet formation 6 months later, but clear effects were found after 1 year [24,25]. One of the reasons for the lack of an effect after 6 months could be that the extent of weight reduction was less pronounced as in our study; i.e., the participants lost only 20% after half a year in the German study, while the BWs decreased in the present investigation by 27.5%. We found in the literature only few studies concerning non-surgical weight loss, and the reduction in the BWs in all investigations was less pronounced. In two SCGE studies, a clear decrease in comet formation was detected after 6 months [59,60], while no evidence for a reduction in the micronucleus frequencies (reflecting structural and numerical chromosomal aberrations) was observed in lymphocytes of individuals after consumption of a low carbohydrate/low protein diet by Benassi-Evans and co-workers [61].

It is known that the formation of “comets” reflects adverse health effects in humans, i.e., a recent analysis showed that “large comets” in humans are indicative for increased mortality [12]. Furthermore, it is well documented that subjects with oxidative stress (due to diabetes and other diseases) have more DNA damage [62], and it is also well documented that exposures to chemicals and radiation, which lead to cancer, cause DNA migration [63]. On the other hand, reduction of comet formation may be indicative of positive effects. In this context it is notable that plant-derived foods, beverages, vitamins, and trace elements with cancer protective and antioxidant properties reduce comet formation in humans [34].

We did not detect significant reduction of FPG-sensitive sites which reflect formation of oxidized purines, but their formation decreased in a time-dependent manner in the overall BS group. We found this effect also in the OAGB group. It was stated recently by the EFSA that prevention of oxidative DNA damage has beneficial health consequences [14]. Several earlier articles concern the oxidation of guanosine after BS. For example, Monzo-Beltran and co-workers [28] reported a decline of 8-oxodG in serum and urine samples after GS at time points ≥ 6 months; the same observation was also made in a Turkish study [27]. Carlsson and co-workers [26] measured 8-oxodG and 8-oxoGuo in urine samples after RYGB surgery; both markers decreased 1–2 years after the surgery, while an increase in oxidative DNA damage was detected 3 months after the operations. This observation is interesting as we observed in RYGB patients a pronounced increase (by 29%) of FPG sensitive sites after 1 month, possibly as a consequence of post-surgical redox stress (data not shown); notably, no such effect was observed in the OAGB group.

BER and NER are prominent repair pathways in eukaryotic organisms [15] and dysfunctions lead to fatal diseases such as cancer and accelerate aging [15,64]. The impact of BS on the activities of these repair systems was not studied earlier. We found in the present study a clear time dependent decrease in the activities of BER and NER regardless of the type of surgery. These findings were unexpected as it was found in earlier human obesity studies that the activities of both repair systems are higher in lean individuals [9,65]. An explanation for the lower levels which we found after weight loss is the intake of a dietary supplement containing vitamins and trace elements. Supplements are given routinely to BS patients since the uptake of micronutrients is reduced after the operations [35,66,67]. It was found in earlier studies that DNA repair functions decrease after consumption of supplements and antioxidant rich foods. For example, reduction in BER was observed after intake of folate [68]. Additionally, after consumption of a vitamin supplement and an antioxidant rich diet a decrease in this repair system was observed [69]. A decrease in NER was reported in a study with kiwi fruit and also after consumption of antioxidant-rich plant products [70]. According to the authors, these effects may be due to adaptive responses, i.e., downregulation as a consequence of lower levels of DNA damage. It is interesting that we found alterations of the repair systems already 1 month after the surgery. These effects increased only moderately in the following months. On the contrary, DNA damage decreased only slightly after the first month and much stronger effects were seen at the last time point. These differences in the time kinetics indicate that weight loss is not the cause for the alterations of the repair systems.

It is difficult to elucidate which molecular mechanisms account for the reduction of oxidative DNA damage, which we observed. BS had in our study no impact on the activities of SOD and GPx, which are important health related antioxidant enzymes. The expression of genes which encode for these enzymes is regulated by the transcription factor Nrf2, and evidence for its activation was observed in a previous study with BS patients [25]. Results of earlier investigations on the activities of these enzymes after BS are controversial. Guan et al. [71] found no alterations of SOD in GS and RYGB patients after 6 months and an increase in the latter group after 12 months. Abad-Jimenez [32] reported an increase in both enzymes after RYGB surgery, while other investigations found reduced activities [31,72]. Many earlier studies indicated that reduction in body weight leads to normalization of the glucose and insulin levels [9,73], and it is well document that increased concentrations lead to release of ROS and cause damage of the genetic material under in vitro conditions (for details, see [9,74]), and also the levels of triglycerides, which are elevated in obese individuals, may play a causal role. It was found in an earlier study that their levels correlate with the extent of DNA damage in obese individuals [75]. A further possible explanation of the high levels of DNA damage as a consequence of excess overweight is the formation of lipid peroxidation (LP) products, which are formed as a consequence of oxidation of fatty acids. Many products of this reaction (aldehydes and ketones) cause DNA damage and cancer. As described in the results section, we found a pronounced decrease in the formation of MDA after BS in the present study. This observation was not unexpected as it is known that MDA levels and other LP products are increased in obese individuals as a consequence of oxidative stress [76,77,78]. In this context, it is notable that several aldehydes and ketones which are formed as a consequence of the oxidation of fatty acids cause DNA damage and cancer [23].

The impact of BS on telomere lengths is a controversial issue. We analyzed in the present study alterations of telomere lengths by two assays using two different single-copy genes ALB and 36B4. The ALB assay showed an increase in telomere lengths between one and 6 months in the overall group and in the OAGB subgroup. In support of this outcome, the 36B4 assay was increased at the end of the study, but this effect did not reach significance. Results of earlier investigations are described in a review of Pene and co-workers [30]. The authors concluded that only results of long-term studies suggest a clear effect on telomere lengths and stated that it is difficult to drawn firm conclusions. Additionally, reviews concerning the consequence of non-surgical weight loss on telomere lengths point in the direction of positive long-term effects [79,80].

Proteome profiling identified a number of proteins, which were altered after BS. Clear upregulation of APOA4, which is indicative for changes of the lipid metabolism, was also found in earlier studies [81]. In the present study, four proteins were downregulated after 6 months. The decrease in beta globulin and alpha globulin is probably a consequence of iron deficiency, which was observed in earlier BS studies [82]. SAA1 and CRP are biomarkers of acute inflammation and cancer development [83,84]; also, in earlier proteome analyses of BS patients, a decrease in the concentrations of both proteins was reported [81,85].

## 5. Conclusions

The results of the present study show that BS leads to stabilization of the genetic material and reduces oxidative DNA damage; these findings are possibly causally related to a decrease in inflammatory reactions. Our findings indicate that adverse health effects, which are caused by increased BW as a consequence of instability of the genetic material, can be reduced by BS.

## Figures and Tables

**Table 1:** Demographic data of the patients (*n* = 35).

Characteristics	Values ^1^
**Average age (years)**	
All	41.3 ± 13.3
OAGB	40.1 ± 12.2
RYGB	43.6 ± 13.9
GS	50.5 ± 10.6
SADI-S	33.3 ± 20.0
**Gender**	
All	29 F, 6 M
OAGB	18 F, 1 M
RYGB	8 F, 3 M
GS	2 M
SADI-S	3 F
**Smoking**	
All	13 (12 F, 1 M)
OAGB	5 (5 F)
RYGB	4 (4 F)
GS	1 (1 M)
SADI-S	3 (3 F)
**Initial weight (kg)**	
All	125.5 ± 19.6
OAGB	120.0 ± 12.8
RYGB	128.6 ± 23.5
GS	130.5 ± 13.4
SADI-S	145.3 ± 35.2
**Initial BMI (kg/m^2^)**	
All	45.4 ± 6.6
OAGB	43.6 ± 4.3
RYGB	47.1 ± 8.0
GS	40.2 ± 2.2
SADI-S	54.1 ± 7.5

^1^ Four bariatric techniques were used, namely, OAGB (*n* = 19), RYGB (*n* = 11), GS (*n* = 2), and SADI-S (*n* = 3). Data are presented as means ± SD. Abbreviations: F, females; GS, gastric sleeve; M, male; NS, non-smokers; OAGB, one-anastomosis gastric bypass; RYGB, Roux-en-Y gastric bypass; S, smokers; SADI-S, single-anastomosis duodeno-Ileal bypass.

**Table 2:** Alterations of the activities of antioxidant enzymes in patients (*n* = 35) undergoing bariatric surgery ^1^.

Parameters	before Surgery	1 Month after Surgery	∆ (%) (T0 vs. 1M)	*p*-Values (T0 vs. 1M)	6 Months after Surgery	∆ (%) (T0 vs. 6M)	*p*-Values	∆ (%) (1M vs. 6M)	*p*-Values
**SOD**									
(% inhibition of tetrazolium salt formation)									
All (*n* = 35)	55.8 ± 9.6	52.2 ± 9.4	−6.4	0.158	54.7 ± 8.5	−1.9	0.562	+4.7	0.244
OAGB (*n* = 19)	54.3 ± 9.4	52.3 ± 10.5	−3.6	0.653	53.7 ± 8.5	−1.1	0.854	+2.6	0.694
**GPx** (mU/mL)									
All (*n* = 35)	719.5 ± 163.6	667.0 ± 132.6	−7.3	0.228	701.1 ± 139.5	−2.5	0.787	+5.0	0.151
OAGB (*n* = 19)	690.9 ± 168.0	642.7 ± 116.9	−6.9	0.437	669.2 ± 123.2	+1.2	0.825	+4.1	0.407

Abbreviations: GPx, glutathione peroxidase; OAGB, one-anastomosis gastric bypass; SOD, superoxide dismutase. ^1^ Results are presented as means ± SD. All samples were measured in duplicate. *p*-values were calculated with ANOVA with Bonferroni correction of linear contrasts (gender and age as covariates). Negative values indicate a decrease in the activities of antioxidant enzymes, and positive values indicate an increase.

**Table 3:** Telomere lengths before and after bariatric surgery ^1^.

Telomere (T/S Ratio) (in Lymphocytes)	before Surgery	1 Month after Surgery	∆ (%) (T0 vs. 1M)	*p*-Values	6 Months after Surgery	∆ (%) (T0 vs. 6M)	*p*-Values	∆ (%) (1M vs. 6M)	*p*-Values
**relTL-ALB**									
All (*n* = 34) ^2^	1.23 ± 1.19	0.99 ± 0.46	−19.5	0.353	1.11 ± 0.42	−9.7	0.512	+12.1	0.022 *
OAGB (*n* = 19)	1.24 ± 0.83	1.00 ± 0.39	−19.3	0.157	1.18 ± 0.44	−4.8	0.712	+18.0	0.046 *
**relTL-36B4**									
All (*n* = 34) ^2^	1.05 ± 0.47	1.02 ± 0.44	−2.8	0.797	1.08 ± 0.43	+2.8	0.447	+5.8	0.300
OAGB (*n* = 19)	1.16 ± 0.47	1.05 ± 0.44	−9.4	0.220	1.18 ± 0.38	+1.7	0.667	+12.3	0.125

Abbreviations: OAGB, one-anastomosis gastric bypass; relTL, relative telomere length ^1^ Results are presented as means ± SD, *p*-values were calculated with ANOVA and Bonferroni correction of linear contrasts (gender and age as covariates). Asterisks indicate statistical significance (* *p* ≤ 0.05). Negative values reflect a decrease in telomere lengths, positive values an increase. ^2^ *n* = 34; in one subject the number of lymphocytes was not sufficiently high.

## Data Availability

Data available on request due to restrictions e.g., privacy or ethical.

## Supplementary Materials

The following supporting information can be downloaded at: https://www.mdpi.com/article/10.3390/antiox12030760/s1, Figure S1: Impact of bariatric surgery on DNA stability (2A), oxidative DNA damage (2B) and DNA repair (2C,D); Figure S2: Impact of bariatric surgery on the activities of the antioxidant enzymes SOD (3A) and GPx (3B); Table S1: Dietary supplements after bariatric surgery; Table S2: List of analyzed proteins.

## Abbreviations

ACN	acetonitrile
APOA4	apolipoprotein A-IV
BCA	bicinchoninic acid assay
BER	base excision repair
BMI	body mass index
BS	bariatric surgery
BW	body weight
CI	confidence interval
CRP	C-reactive protein
EFSA	European Food Safety Authority
FA	formic acid
FDR	false discovery rate
FPG	formamidopyrimidine glycosylase
GPx	glutathione peroxidase
GS	gastric sleeve
LFQ	label-free quantification
LMPA	low melting point agarose
LP	lipid peroxidation
MDA	malondialdehyde
NER	nucleotide excision repair
NMPA	normal melting point agarose
OAGB	one-anastomosis gastric bypass
PASEF	parallel accumulation-serial fragmentation
relTL	relative telomere length
ROS	reactive oxygen species
RYGB SAA1	Roux-en-Y gastric bypass serum amyloid A1
SADI-S	single-anastomosis duodeno-ileal bypass with sleeve gastrectomy
SC	standard conditions
SCGE	single-cell gel electrophoresis
SD	standard deviation
SOD	superoxide dismutase
