# Supplementary material for: Impact of Bariatric Surgery on the Stability of the Genetic Material, Oxidation, and Repair of DNA and Telomere Lengths
Source: Antioxidants (Basel). 2023 Mar 21;12(3):760. doi: 10.3390/antiox12030760 (PMC10045389; doi:10.3390/antiox12030760)
Supplement: Supplementary file 1 [file antioxidants-12-00760-s001.zip › antioxidants-2288769-supplementary.pdf]

Figures S1 A-D

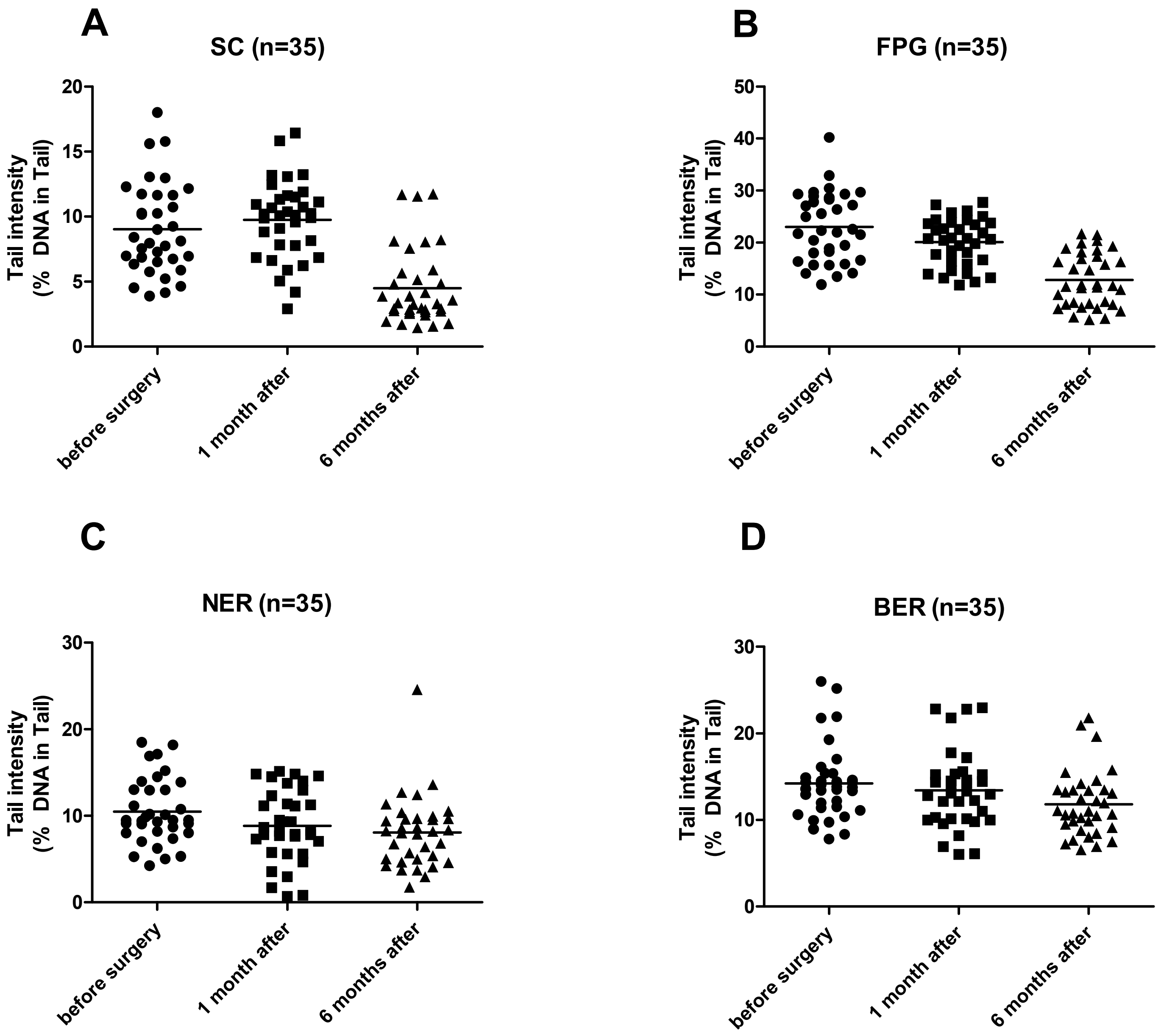

Figures S1 A-D. Impact of bariatric surgery on DNA stability (2A), oxidative DNA damage (2B) and DNA repair (2C-D). Points indicate results which measured with individual samples. In experiments concerning formation of FPG sensitive sites the raw values are indicated which were obtained with enzyme plus buffer. In the case of DNA repair measurements, values are indicated which were obtained with individual samples. Reference values measured with UV (NER) and Ro 19-8023 (BER) were subtracted.

Figure S2 A-B

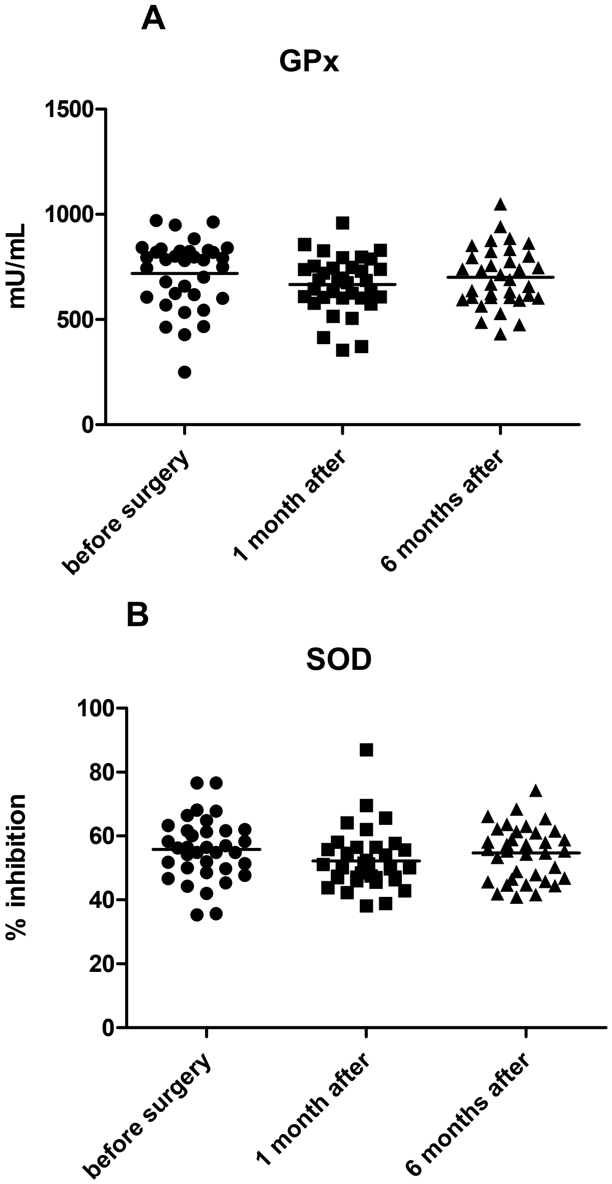

Figure S2 A-B. Impact of bariatric surgery on the activities of the antioxidant enzymes GPx (A) and SOD (B). Points represented individual values which were obtained with individual samples.

Table S1 Dietary supplements after bariatric surgery[1]

Vitamins	Chemical compounds	Amount	RI[2]
Vitamin A	Retinyl palmitate	600 µg RE (1998 IU)	75%
Vitamin B1	Thiamine HCL	2,75 mg	250%
Vitamin B2	Riboflavin	2 mg	143%
Niacin (B3)	Nicotinamide	32 mg NE	200%
Pantothenic acid (B5)	Calcium pantothenate	18 mg	300%
Vitamin B6	Pyridoxal-5′-phosphate	0,98 mg	70%
Biotin (B8)	Biotin	100 µg	200%
Folic acid (B11)	Pteroylmonoglutamic acid	600 µg	300%
Vitamin B12	Cyanocobalamin	350 µg	14000%
Vitamin C	Ascorbic acid	120 mg	150%
Vitamin D3	Cholecalciferol	75 µg (3000 IU)	1500%
Vitamin E	d-alpha-Tocopherol succinat	24 mg α-ET (36 IU)	200%
**Minerals**			
Chromium	Chromium III chloride	160 µg	400%
Copper	Copper gluconate	3 mg	300%
Iron	Ferric fumarate	70 mg	500%
Iodide	Potasium iodide	150 µg	100%
Manganese	Manganese citrate	3 mg	150%
Molybdenum	Sodium molybdate	112,4 µg	225%
Selenium	Sodium selenite	105 µg	191%
Zinc	Zink citrate	22,5 mg	225%

[1] WLS forte (Berlin, Germany) should be taken as one pill per day.
[2] RI, reference intake values based on scientific advice from the European Food Safety Authority (EFSA).

Table S2 List of analyzed proteins

Uniprot Accession	Protein names	Gene names	acute phase response
P62258	14-3-3 protein zeta/delta	*YWHAZ*	
P63104	6-phosphogluconate dehydrogenase, decarboxylating	*PGD*	
P52209	Actin, alpha skeletal muscle	*ACTA1*	
P68133	Actin, cytoplasmic 1	*ACTB*	
P60709	Actin, cytoplasmic 2	*ACTG1*	
P63261	Actin-related protein 2/3 complex subunit 3	*ARPC3*	
O15145	Actin-related protein 3	*ACTR3*	
P61158	Adenylyl cyclase-associated protein 1	*CAP1*	
Q01518	Adiponectin	*ADIPOQ*	
Q15848	Afamin	*AFM*	
P43652	Alcohol dehydrogenase 1B	*ADH1B*	
P00325	Alpha-1-acid glycoprotein 1	*ORM1*	**X**
P02763	Alpha-1-acid glycoprotein 2	*ORM2*	**X**
P19652	Alpha-1-antichymotrypsin	*SERPINA3*	**X**
P01011	Alpha-1-antitrypsin	*SERPINA1*	**X**
P01009	Alpha-1B-glycoprotein	*A1BG*	
P04217	Alpha-2-antiplasmin	*SERPINF2*	**X**
P08697	Alpha-2-HS-glycoprotein	*AHSG*	**X**
P02765	Alpha-2-macroglobulin	*A2M*	**X**
P01023	Alpha-2-macroglobulin-like protein 1	*A2ML1*	
A8K2U0	Alpha-actinin-1	*ACTN1*	
P12814	Alpha-actinin-4	*ACTN4*	
O43707	Alpha-enolase	*ENO1*	
P06733	Angiotensinogen	*AGT*	
P01019	Annexin A1	*ANXA1*	
P04083	Annexin A2	*ANXA2*	
P07355	Annexin A3	*ANXA3*	
P12429	Antileukoproteinase	*SLPI*	
P03973	Antithrombin-III	*SERPINC1*	
P01008	Apolipoprotein A-I	*APOA1*	
P02647	Apolipoprotein A-II	*APOA2*	
P02652	Apolipoprotein A-IV	*APOA4*	
P06727	Apolipoprotein B-100	*APOB*	
P04114	Apolipoprotein C-I	*APOC1*	
P02654	Apolipoprotein C-II	*APOC2*	
P02655	Apolipoprotein C-III	*APOC3*	
P02656	Apolipoprotein C-IV	*APOC4*	
P55056	Apolipoprotein D	*APOD*	
P05090	Apolipoprotein E	*APOE*	
P02649	Apolipoprotein F	*APOF*	

Uniprot Accession	Protein names	Gene names	acute phase response
O14791	Apolipoprotein L1	*APOL1*	
O95445	Apolipoprotein M	*APOM*	
P08519	Apolipoprotein(a)	*LPA*	
P05089	Arginase-1	*ARG1*	
P06576	ATP synthase subunit beta, mitochondrial	*ATP5B*	
O75882	Attractin	*ATRN*	
P17213	Bactericidal permeability-increasing protein	*BPI*	
P02749	Beta-2-glycoprotein 1	*APOH*	
P61769	Beta-2-microglobulin	*B2M*	
Q96KN2	Beta-Ala-His dipeptidase	*CNDP1*	
Q9HBI1	Beta-parvin	*PARVB*	
P52849	Bifunctional heparan sulfate N-deacetylase/N-sulfotransferase 2	*NDST2*	
P43251	Biotinidase	*BTD*	
Q13867	Bleomycin hydrolase	*BLMH*	
Q9NP55	BPI fold-containing family A member 1	*BPIFA1*	
Q8TDL5	BPI fold-containing family B member 1	*BPIFB1*	
P04003	C4b-binding protein alpha chain	*C4BPA*	
P20851	C4b-binding protein beta chain	*C4BPB*	
Q9NZT1	Calmodulin-like protein 5	*CALML5*	
P00915	Carbonic anhydrase 1	*CA1*	
Q96IY4	Carboxypeptidase B2	*CPB2*	
P15169	Carboxypeptidase N catalytic chain	*CPN1*	
P22792	Carboxypeptidase N subunit 2	*CPN2*	
P31944	Caspase-14	*CASP14*	
P04040	Catalase	*CAT*	
P07858	Cathepsin B	*CTSB*	
P07339	Cathepsin D	*CTSD*	
P08311	Cathepsin G	*CTSG*	
O43866	CD5 antigen-like	*CD5L*	
P00450	Ceruloplasmin	*CP*	
O00299	Chloride intracellular channel protein 1	*CLIC1*	
P06276	Cholinesterase	*BCHE*	
Q12873	Chromodomain-helicase-DNA-binding protein 3	*CHD3*	
P10909	Clusterin	*CLU*	
P00740	Coagulation factor IX	*F9*	
P00742	Coagulation factor X	*F10*	
P03951	Coagulation factor XI	*F11*	
P00748	Coagulation factor XII	*F12*	
P00488	Coagulation factor XIII A chain	*F13A1*	X
P05160	Coagulation factor XIII B chain	*F13B*	X
P23528	Cofilin-1	*CFL1*	
Q8WVM7	Cohesin subunit SA-1	*STAG1*	
Q49A88	Coiled-coil domain-containing protein 14	*CCDC14*	
P02745	Complement C1q subcomponent subunit A	*C1QA*	
P02746	Complement C1q subcomponent subunit B	*C1QB*	

Uniprot Accession	Protein names	Gene names	acute phase response
P02747	Complement C1q subcomponent subunit C	*C1QC*	
P00736	Complement C1r subcomponent	*C1R*	
Q9NZP8	Complement C1r subcomponent-like protein	*C1RL*	
P09871	Complement C1s subcomponent	*C1S*	
P06681	Complement C2	*C2*	
P01024	Complement C3	*C3*	
P0C0L4	Complement C4-A	*C4A*	
P0C0L5	Complement C4-B	*C4B*	
P01031	Complement C5	*C5*	
P13671	Complement component C6	*C6*	
P10643	Complement component C7	*C7*	
P07357	Complement component C8 alpha chain	*C8A*	
P07358	Complement component C8 beta chain	*C8B*	
P07360	Complement component C8 gamma chain	*C8G*	
P02748	Complement component C9	*C9*	
P00751	Complement factor B	*CFB*	
P00746	Complement factor D	*CFD*	
P08603	Complement factor H	*CFH*	
Q03591	Complement factor H-related protein 1	*CFHR1*	
P36980-2	Complement factor H-related protein 2	*CFHR2*	
P05156	Complement factor I	*CFI*	
P31146	Coronin-1A	*CORO1A*	
P08185	Corticosteroid-binding globulin	*SERPINA6*	
P02741	C-reactive protein	*CRP*	**X**
P01040	Cystatin-A	*CSTA*	
P01034	Cystatin-C	*CST3*	
P16410	Cytotoxic T-lymphocyte protein 4	*CTLA4*	
Q9UGM3	Deleted in malignant brain tumors 1 protein	*DMBT1*	
P81605	Dermcidin	*DCD*	
Q08554	Desmocollin-1	*DSC1*	
Q02413	Desmoglein-1	*DSG1*	
P15924	Desmoplakin	*DSP*	
Q12805	EGF-containing fibulin-like extracellular matrix protein 1	*EFEMP1*	
P68104	Elongation factor 1-alpha 1	*EEF1A1*	
P13639	Elongation factor 2	*EEF2*	
P58107	Epiplakin	*EPPK1*	
P27105	Erythrocyte band 7 integral membrane protein	*STOM*	
Q16610	Extracellular matrix protein 1	*ECM1*	
P52907	F-actin-capping protein subunit alpha-1	*CAPZA1*	
Q01469	Fatty acid-binding protein, epidermal	*FABP5*	
Q86UX7	Fermitin family homolog 3	*FERMT3*	
P02792	Ferritin light chain	*FTL*	
Q9UGM5	Fetuin-B	*FETUB*	
P02671	Fibrinogen alpha chain	*FGA*	
P02675	Fibrinogen beta chain	*FGB*	

Uniprot Accession	Protein names	Gene names	acute phase response
P02675	Fibrinogen gamma chain	*FGG*	
P02751-14	Fibronectin	*FN1*	X
P23142	Fibulin-1	*FBLN1*	
P23142-4	Fibulin-1	*FBLN1*	
Q15485-2	Ficolin-2	*FCN2*	
O75636	Ficolin-3	*FCN3*	
P21333	Filamin-A	*FLNA*	
P30043	Flavin reductase (NADPH)	*BLVRB*	
P04075	Fructose-bisphosphate aldolase A	*ALDOA*	
Q08380	Galectin-3-binding protein	*LGALS3BP*	
P47929	Galectin-7	*LGALS7*	
P09104	Gamma-enolase	*ENO2*	
P17900	Ganglioside GM2 activator	*GM2A*	
Q96QA5	Gasdermin-A	*GSDMA*	
P06396	Gelsolin	*GSN*	
P15104	Glutamine synthetase	*GLUL*	
P22352	Glutathione peroxidase 3	*GPX3*	
P09211	Glutathione S-transferase P	*GSTP1*	
P04406	Glyceraldehyde-3-phosphate dehydrogenase	*GAPDH*	
P06737	Glycogen phosphorylase, liver form	*PYGL*	
P04899	Guanine nucleotide-binding protein G(i) subunit alpha-2	*GNAI2*	
P00738	Haptoglobin	*HP*	X
P00739	Haptoglobin-related protein	*HPR*	
P0DMV8	Heat shock 70 kDa protein 1A	*HSPA1A*	
P04792	Heat shock protein beta-1	*HSPB1*	
P07900	Heat shock protein HSP 90-alpha	*HSP90AA1*	
P69905	Hemoglobin subunit alpha	*HBA1*	
P68871	Hemoglobin subunit beta	*HBB*	
P02042	Hemoglobin subunit delta	*HBD*	
P69892	Hemoglobin subunit gamma-2	*HBG2*	
P02790	Hemopexin	*HPX*	
P05546	Heparin cofactor 2	*SERPIND1*	
Q04756	Hepatocyte growth factor activator	*HGFAC*	
P26927	Hepatocyte growth factor-like protein	*MST1*	
P09651	Heterogeneous nuclear ribonucleoprotein A1	*HNRNPA1*	
P22626	Heterogeneous nuclear ribonucleoproteins A2/B1	*HNRNPA2B1*	
P52790	Hexokinase-3	*HK3*	
P04196	Histidine-rich glycoprotein	*HRG*	
P16401	Histone H1.5	*HIST1H1B*	
P0C0S8	Histone H2A type 1	*HIST1H2AG*	
Q6FI13	Histone H2A type 2-A	*HIST2H2AA3*	
Q99880	Histone H2B type 1-L	*HIST1H2BL*	
P62805	Histone H4	*HIST1H4A*	
Q30167	HLA class II histocompatibility antigen, DRB1-10 beta chain	*HLA-DRB1*	
Q14520	Hyaluronan-binding protein 2	*HABP2*	

Uniprot Accession	Protein names	Gene names	acute phase response
P01876	Ig alpha-1 chain C region	*IGHA1*	
P0DOX2	Ig alpha-2 chain C region	*IGHA2*	
P01880	Ig delta chain C region	*IGHD*	
P0DOX5	Ig gamma-1 chain C region	*IGHG1*	
P01859	Ig gamma-2 chain C region	*IGHG2*	
P01860	Ig gamma-3 chain C region	*IGHG3*	
P01861	Ig gamma-4 chain C region	*IGHG4*	
P01742	Ig heavy chain V-I region EU	*IGHV1-69*	
P01743	Ig heavy chain V-I region HG3	*IGHV1-46*	
P23083	Ig heavy chain V-I region V35	*IGHV1-2*	
P06331	Ig heavy chain V-II region ARH-77	*IGHV4-34*	
P01817	Ig heavy chain V-II region MCE	*IGHV2-5*	
A0A0C4DH41	Ig heavy chain V-II region NEWM	*IGHV4-61*	
P01814	Ig heavy chain V-II region OU	*IGHV2-70*	
P01824	Ig heavy chain V-II region WAH	*IGHV4-39*	
P01764	Ig heavy chain V-III region 23	*IGHV3-23*	
P01766	Ig heavy chain V-III region BRO	*IGHV3-13*	
P01767	Ig heavy chain V-III region BUT	*IGHV3-7*	
P01782	Ig heavy chain V-III region DOB	*IGHV3-9*	
P01780	Ig heavy chain V-III region JON	*IGHV3-7*	
P01762	Ig heavy chain V-III region TRO	*IGHV3-11*	
P01763	Ig heavy chain V-III region WEA	*IGHV3-48*	
P01834	Ig kappa chain C region	*IGKC*	
P01594	Ig kappa chain V-I region AU	*IGKV1-33*	
P04430	Ig kappa chain V-I region BAN	*IGKV1-16*	
P04432	Ig kappa chain V-I region Daudi	*IGKV1D-39*	
P01599	Ig kappa chain V-I region Gal	*IGKV1-17*	
P01601	Ig kappa chain V-I region HK101	*IGKV1-39*	
P01602	Ig kappa chain V-I region HK102	*IGKV1-5*	
A0A0C4DH73	Ig kappa chain V-I region Wes	*IGKV1-12*	
P01614	Ig kappa chain V-II region Cum	*IGKV2-40*	
A0A075B6P5	Ig kappa chain V-II region FR	*IGKV2D-28*	
P06310	Ig kappa chain V-II region RPMI 6410	*IGKV2D-30*	
P01619	Ig kappa chain V-III region B6	*IGKV3-20*	
P04433	Ig kappa chain V-III region VG	*IGKV3D-11*	
P06312	Ig kappa chain V-IV region	*IGKV4-1*	
P01700	Ig lambda chain V-I region HA	*IGLV1-47*	
P01701	Ig lambda chain V-I region NEW	*IGLV1-51*	
P01703	Ig lambda chain V-I region NEWM	*IGLV1-40*	
P01699	Ig lambda chain V-I region VOR	*IGLV1-44*	
P01706	Ig lambda chain V-II region BOH	*IGLV2-11*	
P01704	Ig lambda chain V-II region TOG	*IGLV2-14*	
P80748	Ig lambda chain V-III region LOI	*IGLV3-21*	
P01714	Ig lambda chain V-III region SH	*IGLV3-19*	
P01715	Ig lambda chain V-IV region Bau	*IGLV3-1*	

Uniprot Accession	Protein names	Gene names	acute phase response
P01718	Ig lambda chain V-IV region Kern	*IGLV3-27*	
P01721	Ig lambda chain V-VI region AR	*IGLV6-57*	
P0DOY3	Ig lambda-6 chain C region	*IGLC6*	
P01871	Ig mu chain C region	*IGHM*	
P01591	Immunoglobulin J chain	*IGJ*	
P15814	Immunoglobulin lambda-like polypeptide 1	*IGLL1*	
B9A064	Immunoglobulin lambda-like polypeptide 5	*IGLL5*	
O14732	Inositol monophosphatase 2	*IMPA2*	
P17936	Insulin-like growth factor-binding protein 3	*IGFBP3*	
P35858	Insulin-like growth factor-binding protein complex acid labile subunit	*IGFALS*	
P08514	Integrin alpha-IIb	*ITGA2B*	
P19827	Inter-alpha-trypsin inhibitor heavy chain H1	*ITIH1*	
P19823	Inter-alpha-trypsin inhibitor heavy chain H2	*ITIH2*	
Q06033	Inter-alpha-trypsin inhibitor heavy chain H3	*ITIH3*	
Q14624	Inter-alpha-trypsin inhibitor heavy chain H4	*ITIH4*	**X**
P18510-4	Interleukin-1 receptor antagonist protein	*IL1RN*	
P14923	Junction plakoglobin	*JUP*	
P29622	Kallistatin	*SERPINA4*	
Q5T749	Keratinocyte proline-rich protein	*KPRP*	
Q96Q89	Kinesin-like protein KIF20B	*KIF20B*	
P01042	Kininogen-1	*KNG1*	
P01042-2	Kininogen-1	*KNG1*	
P02788	Lactotransferrin	*LTF*	
P02750	Leucine-rich alpha-2-glycoprotein	*LRG1*	
P30740	Leukocyte elastase inhibitor	*SERPINB1*	
P09960	Leukotriene A-4 hydrolase	*LTA4H*	
Q9UPQ0-3	LIM and calponin homology domains-containing protein 1	*LIMCH1*	
P31025	Lipocalin-1	*LCN1*	
P18428	Lipopolysaccharide-binding protein	*LBP*	**X**
P00338	L-lactate dehydrogenase A chain	*LDHA*	
P07195	L-lactate dehydrogenase B chain	*LDHB*	
O75015	Low affinity immunoglobulin gamma Fc region receptor III-B	*FCGR3B*	
P51884	Lumican	*LUM*	
P61626	Lysozyme C	*LYZ*	
P48740	Mannan-binding lectin serine protease 1	*MASP1*	
O00187	Mannan-binding lectin serine protease 2	*MASP2*	
Q96L34	MAP/microtubule affinity-regulating kinase 4	*MARK4*	
P14780	Matrix metalloproteinase-9	*MMP9*	
P26038	Moesin	*MSN*	
P08571	Monocyte differentiation antigen CD14	*CD14*	
Q9HC84	Mucin-5B	*MUC5B*	
P24158	Myeloblastin	*PRTN3*	
P41218	Myeloid cell nuclear differentiation antigen	*MNDA*	
P05164	Myeloperoxidase	*MPO*	
P60660	Myosin light polypeptide 6	*MYL6*	

Uniprot Accession	Protein names	Gene names	acute phase response
Q7Z406	Myosin-14	*MYH14*	
P35579	Myosin-9	*MYH9*	
Q8NEV4	Myosin-IIIa	*MYO3A*	
Q96PD5	N-acetylmuramoyl-L-alanine amidase	*PGLYRP2*	
E9PAV3	Nascent polypeptide-associated complex subunit alpha, muscle-specific form	*NACA*	
P22894	Neutrophil collagenase	*MMP8*	
P14598	Neutrophil cytosol factor 1	*NCF1*	
P80188	Neutrophil gelatinase-associated lipocalin	*LCN2*	
P43490	Nicotinamide phosphoribosyltransferase	*NAMPT*	
Q6UX06	Olfactomedin-4	*OLFM4*	
P04746	Pancreatic alpha-amylase	*AMY2A*	
P62937	Peptidyl-prolyl cis-trans isomerase A	*PPIA*	
P32119	Peroxiredoxin-2	*PRDX2*	
P30044	Peroxiredoxin-5, mitochondrial	*PRDX5*	
P80108	Phosphatidylinositol-glycan-specific phospholipase D	*GPLD1*	
P00558	Phosphoglycerate kinase 1	*PGK1*	
P18669	Phosphoglycerate mutase 1	*PGAM1*	
P55058	Phospholipid transfer protein	*PLTP*	
P36955	Pigment epithelium-derived factor	*SERPINF1*	
P03952	Plasma kallikrein	*KLKB1*	
P05155	Plasma protease C1 inhibitor	*SERPING1*	
P05154	Plasma serine protease inhibitor	*SERPINA5*	
P00747	Plasminogen	*PLG*	
P13796	Plastin-2	*LCP1*	
P02775	Platelet basic protein	*PPBP*	
P02776	Platelet factor 4	*PF4*	
P08567	Pleckstrin	*PLEK*	
P01833	Polymeric immunoglobulin receptor	*PIGR*	
P0CG48	Polyubiquitin-C	*UBC*	
P20742	Pregnancy zone protein	*PZP*	
P51531	Probable global transcription activator SNF2L2	*SMARCA2*	
P07737	Profilin-1	*PFN1*	
P12273	Prolactin-inducible protein	*PIP*	
P41222	Prostaglandin-H2 D-isomerase	*PTGDS*	
P02760	Protein AMBP	*AMBP*	
O75629	Protein CREG1	*CREG1*	
Q8WVV4-1	Protein POF1B	*POF1B*	
P31949	Protein S100-A11	*S100A11*	
P80511	Protein S100-A12	*S100A12*	
P06703	Protein S100-A6	*S100A6*	
P31151	Protein S100-A7	*S100A7*	
P05109	Protein S100-A8	*S100A8*	
P06702	Protein S100-A9	*S100A9*	
P25815	Protein S100-P	*S100P*	
Q9UK55	Protein Z-dependent protease inhibitor	*SERPINA10*	

Uniprot Accession	Protein names	Gene names	acute phase response
Q9UM07	Protein-arginine deiminase type-4	*PADI4*	
Q08188	Protein-glutamine gamma-glutamyltransferase E	*TGM3*	
P22735	Protein-glutamine gamma-glutamyltransferase K	*TGM1*	
Q92954	Proteoglycan 4	*PRG4*	
P00734	Prothrombin	*F2*	
Q17R31	Putative deoxyribonuclease TATDN3	*TATDN3*	
A6NCN2	Putative keratin-87 protein	*KRT87P*	
Q76L83	Putative Polycomb group protein ASXL2	*ASXL2*	
P14618	Pyruvate kinase PKM	*PKM*	
P50395	Rab GDP dissociation inhibitor beta	*GDI2*	
P61224	Ras-related protein Rap-1b	*RAP1B*	
P02753	Retinol-binding protein 4	*RBP4*	
Q53RT3	Retroviral-like aspartic protease 1	*ASPRV1*	
P52565	Rho GDP-dissociation inhibitor 1	*ARHGDIA*	
P52566	Rho GDP-dissociation inhibitor 2	*ARHGDIB*	
P49908	Selenoprotein P	*SEPP1*	
P04279	Semenogelin-1	*SEMG1*	
Q02383	Semenogelin-2	*SEMG2*	
O75460	Serine/threonine-protein kinase/endoribonuclease IRE1	*ERN1*	
O15084	Serine/threonine-protein phosphatase 6 regulatory ankyrin repeat subunit A	*ANKRD28*	
P02787	Serotransferrin	*TF*	
Q96P63	Serpin B12	*SERPINB12*	
P29508	Serpin B3	*SERPINB3*	
P48594	Serpin B4	*SERPINB4*	
P0DJI8	Serum amyloid A-1 protein	*SAA1*	
P0DJI9	Serum amyloid A-2 protein	*SAA2*	
P35542	Serum amyloid A-4 protein	*SAA4*	
P02743	Serum amyloid P-component	*APCS*	
P27169	Serum paraoxonase/arylesterase 1	*PON1*	
P04278	Sex hormone-binding globulin	*SHBG*	
Q9UBC9	Small proline-rich protein 3	*SPRR3*	
A0AV02	Solute carrier family 12 member 8	*SLC12A8*	
P02808	Statherin	*STATH*	
O00391	Sulfhydryl oxidase 1	*QSOX1*	
Q9Y490	Talin-1	*TLN1*	
O14981	TATA-binding protein-associated factor 172	*BTAF1*	
P10599	Thioredoxin	*TXN*	
P05543	Thyroxine-binding globulin	*SERPINA7*	
P37837	Transaldolase	*TALDO1*	
Q15582	Transforming growth factor-beta-induced protein ig-h3	*TGFBI*	
P37802	Transgelin-2	*TAGLN2*	
P29401	Transketolase	*TKT*	
P02766	Transthyretin	*TTR*	
P60174	Triosephosphate isomerase	*TPI1*	
O14773	Tripeptidyl-peptidase 1	*TPP1*	

Uniprot Accession	Protein names	Gene names	acute phase response
P67936	Tropomyosin alpha-4 chain	*TPM4*	
P68363	Tubulin alpha-1B chain	*TUBA1B*	
P68366	Tubulin alpha-4A chain	*TUBA4A*	
P07437	Tubulin beta chain	*TUBB*	
P08670	Vimentin	*VIM*	
P18206	Vinculin	*VCL*	
P02774	Vitamin D-binding protein	*GC*	
P04070	Vitamin K-dependent protein C	*PROC*	
P07225	Vitamin K-dependent protein S	*PROS1*	
P04004	Vitronectin	*VTN*	
P04275	von Willebrand factor	*VWF*	
O75083	WD repeat-containing protein 1	*WDR1*	
P25311	Zinc-alpha-2-glycoprotein	*AZGP1*	
Q96DA0	Zymogen granule protein 16 homolog B	*ZG16B*	
A0A075B6H7	Immunoglobulin kappa variable 3-7 (Non-Functional)	*IGKV3-7*	
A0A075B6K2	Immunoglobulin lambda variable 3-12	*IGLV3-12*	
A0A075B6K4	Immunoglobulin lambda variable 3-10	*IGLV3-10*	
A0A075B6K5	Immunoglobulin lambda variable 3-9	*IGLV3-9*	
A0A075B6R2	Immunoglobulin heavy variable 4-4	*IGHV4-4*	
A0A075B6S2	Immunoglobulin kappa variable 2D-29	*IGKV2D-29*	
A0A075B6S5	Immunoglobulin kappa variable 1-27	*IGKV1-27*	
A0A087WSX0	Immunoglobulin kappa variable 5-45	*IGLV5-45*	
A0A087WSY6	Immunoglobulin kappa variable 3D-15	*IGKV3D-15*	
A0A087WSZ0	Immunoglobulin kappa variable 1D-8	*IGKV1D-8*	
A0A0A0MS15	Immunoglobulin heavy variable 3-49	*IGHV3-49*	
A0A0B4J1U3	Immunoglobulin lambda variable 1-36	*IGLV1-36*	
A0A0B4J1U7	Immunoglobulin heavy variable 6-1	*IGHV6-1*	
A0A0B4J1V1	Immunoglobulin heavy variable 3-21	*IGHV3-21*	
A0A0B4J1V2	Immunoglobulin heavy variable 2-26	*IGHV2-26*	
A0A0B4J1Y9	Immunoglobulin heavy variable 3-72	*IGHV3-72*	
A0A0B4J2H0	Immunoglobulin heavy variable 1-69-2	*IGHV1-69-2*	
A0A0C4DH24	Immunoglobulin kappa variable 6-21 (Non-Functional)	*IGKV6-21*	
A0A0C4DH25	Immunoglobulin kappa variable 3D-20	*IGKV3D-20*	
A0A0C4DH29	Immunoglobulin heavy variable 1-3	*IGHV1-3*	
A0A0C4DH31	Immunoglobulin heavy variable 1-18	*IGHV1-18*	
A0A0C4DH33	Immunoglobulin heavy variable 1-24	*IGHV1-24*	
A0A0C4DH34	Immunoglobulin heavy variable 4-28	*IGHV4-28*	
A0A0C4DH35	Immunoglobulin heavy variable 3-35	*IGHV3-35*	
A0A0C4DH36	Immunoglobulin heavy variable 3-38	*IGHV3-38*	
A0A0C4DH38	Immunoglobulin heavy variable 5-51	*IGHV5-51*	
A0A0C4DH55	Immunoglobulin kappa variable 3D-7	*IGKV3D-7*	
A0A0C4DH67	Immunoglobulin kappa variable 1-8	*IGKV1-8*	
A0A0C4DH68	Immunoglobulin kappa variable 2-24	*IGKV2-24*	
A0A0J9YX35	Immunoglobulin heavy variable 3-64D	*IGHV3-64D*	
A2NJV5	Immunoglobulin kappa variable 2-29	*IGKV A18*	

Uniprot Accession	Protein names	Gene names	acute phase response
P0DOX6	Immunoglobulin delta heavy chain	*N/A*	
P0DOX7	Immunoglobulin mu heavy chain	*N/A*	
P0DP01	Immunoglobulin kappa light chain	*N/A*	
P0DP02	Immunoglobulin heavy variable 1-8	*IGHV1-8*	
P0DP09	Immunoglobulin heavy variable 3-30-3	*IGHV3-30-3*	
P0DP25	Immunoglobulin kappa variable 1D-13	*IGKV1D-13*	
P0DSN7	Calmodulin-3	*CALM3*	
Q8WYA6-2	Immunoglobulin kappa variable 1D-37	*IGKV1D-37*	
